# Vaspin in atherosclerotic disease and cardiovascular risk in axial spondyloarthritis: a genetic and serological study

**DOI:** 10.1186/s13075-021-02499-7

**Published:** 2021-04-13

**Authors:** Javier Rueda-Gotor, Raquel López-Mejías, Sara Remuzgo-Martínez, Verónica Pulito-Cueto, Alfonso Corrales, Leticia Lera-Gómez, Virginia Portilla, Íñigo González-Mazón, Ricardo Blanco, Rosa Expósito, Cristina Mata, Javier Llorca, Vanesa Hernández-Hernández, Carlos Rodríguez-Lozano, Nuria Barbarroja, Rafaela Ortega Castro, Esther Vicente, Cristina Fernández-Carballido, María Paz Martínez-Vidal, David Castro-Corredor, Joaquín Anino-Fernández, Diana Peiteado, Chamaida Plasencia-Rodríguez, Eva Galíndez-Agirregoikoa, María Luz García-Vivar, Oreste Gualillo, Juan Carlos Quevedo-Abeledo, Santos Castañeda, Iván Ferraz-Amaro, Miguel Á. González-Gay, Fernanda Genre

**Affiliations:** 1 Research Group on Genetic Epidemiology and Atherosclerosis in Systemic Diseases and in Metabolic Diseases of the Musculoskeletal System, IDIVAL, Hospital Universitario Marqués de Valdecilla, Avenida Cardenal Herrera Oria s/n, Lab. 201/202, 39011 Santander, Spain; 2 Rheumatology Division, Hospital Comarcal de Laredo, Laredo, Spain; 3 Department of Epidemiology and Computational Biology, School of Medicine, University of Cantabria and CIBERESP, Santander, Spain; 4 Rheumatology Division, Hospital Universitario de Canarias, Santa Cruz de Tenerife, Spain; 5 Rheumatology Division, Hospital Universitario de Gran Canaria Dr. Negrín, Las Palmas de Gran Canaria, Spain; 6 Rheumatology Division, Hospital Reina Sofía, Maimonides Institute for Research in Biomedicine of Cordoba (IMIBIC), University of Cordoba, Cordoba, Spain; 7 Rheumatology Division, Hospital Universitario de La Princesa, IIS-Princesa, Madrid, Spain; 8 Rheumatology Division, Hospital Universitario de San Juan, Alicante, Spain; 9 Rheumatology Division, Hospital General Universitario de Alicante, Alicante, Spain; 10 Rheumatology Division, Hospital General Universitario de Ciudad Real, Ciudad Real, Spain; 11 Rheumatology Division, Hospital Universitario La Paz-IdiPaz, Madrid, Spain; 12 Rheumatology Division, Hospital Universitario Basurto, Bilbao, Spain; 13 SERGAS and IDIS, NEIRID Lab, Research Laboratory 9, Santiago University Clinical Hospital, Santiago de Compostela, Spain; 14 School of Medicine, University of Cantabria, Santander, Spain; 15 Cardiovascular Pathophysiology and Genomics Research Unit, School of Physiology, Faculty of Health Sciences, University of the Witwatersrand, Johannesburg, South Africa

**Keywords:** Vaspin, Axial spondyloarthritis, Biomarker, Subclinical atherosclerosis, Cardiovascular risk, Polymorphism

## Abstract

**Background:**

Vaspin is a novel anti-inflammatory adipokine associated with cardiovascular (CV) disease and inflammation in chronic inflammatory conditions different from axial spondyloarthritis (axSpA). Given the high incidence of CV disease (mainly due to accelerated atherosclerosis) exhibited by axSpA patients, we wondered if vaspin could also be a key molecule in this process. However, data on the role of vaspin regarding atherosclerotic disease in the context of axSpA is scarce. For this reason, we aimed to evaluate the implication of vaspin, at the genetic and serological level, in subclinical atherosclerosis and CV risk in axSpA.

**Methods:**

This study included 510 patients diagnosed with axSpA. Carotid ultrasound (US) was performed to evaluate the presence of subclinical atherosclerosis. Three *vaspin* gene variants (rs2236242, rs7159023, and rs35262691) were genotyped by TaqMan probes. Serum vaspin levels were assessed by enzyme-linked immunosorbent assay. Statistical analysis was performed using STATA® v.11.1.

**Results:**

Serum vaspin levels were significantly higher in female patients than in males and also in obese patients when compared to those with normal weight (*p* < 0.05). At the genetic level, we disclosed that the minor allele of rs2236242 (A) was associated with lower serum vaspin levels in axSpA, while the rs7159023 minor allele (A) was linked to higher serum levels (*p* < 0.05). When the three polymorphisms assessed were combined conforming haplotypes, we disclosed that the TGC haplotype related to high serum levels of vaspin (*p* = 0.01). However, no statistically significant association was observed between vaspin and markers of subclinical atherosclerosis, both at the genetic and serological level.

**Conclusions:**

Our results revealed that vaspin is linked to CV risk factors that may influence on the atherosclerotic process in axSpA. Additionally, we disclosed that serum vaspin concentration is genetically modulated in a large cohort of patients with axSpA.

**Supplementary Information:**

The online version contains supplementary material available at 10.1186/s13075-021-02499-7.

## Background

Axial spondyloarthritis (axSpA) is a chronic inflammatory disease that mainly affects the spine and pelvic joints and that prevails in young people [1]. In addition to the clinical manifestations related to the disease, these patients also show a high morbidity and mortality related to cardiovascular (CV) disease, particularly due to a process of accelerated atherosclerosis [2, 3]. The high CV risk observed in axSpA patients is related to an increased prevalence of CV risk factors, chronic systemic inflammation, and a dysregulation in different molecules associated with CV disease [4–7]. Concerning this, the assessment of subclinical atherosclerosis by carotid ultrasound (US) in axSpA patients is becoming very useful to predict CV events [8, 9]. In addition, the combination of this non-invasive imaging technique with the assessment of biomarkers related to CV risk may constitute a major advance in the prevention of CV disease in axSpA.

In previous studies of our group, we assessed the potential role of different molecules as biomarkers of CV risk in axSpA. The results obtained in those studies supported the implication of such molecules in the high CV risk observed in axSpA patients [4, 10, 11]. This led us to search for other molecules that may also be biomarkers of CV risk in axSpA. Consequently, the assessment of a combination of such molecules may be of relevance in the clinical practice to help to predict CV risk in these patients.

A potential candidate biomarker of CV risk in axSpA may be vaspin (visceral adipose tissue-derived serine protease inhibitor), a novel adipokine with insulin-sensitizing functions that exerts anti-inflammatory and anti-atherogenic actions [12]. This protein is coded by the *vaspin* gene (also known as *SERPINA12*) located on chromosome 14q32.13. This gene is expressed in several tissues such as the adipose tissue, skin, stomach, liver, pancreas, and skeletal muscle [12–14]. The multifaceted functions of vaspin seem to be exerted through the induction of intracellular signaling cascades involving AKT, NF-κB, and MAPK [12]. Accordingly, vaspin was associated with CV disease and inflammation in the general population and in chronic inflammatory conditions different from axSpA [15–18]. Given the high incidence of CV disease exhibited by axSpA patients, which turns this comorbidity into a matter of major concern among rheumatologists, we wondered if vaspin may be relevant for axSpA. However, data on the role of vaspin regarding surrogate markers of atherosclerosis and CV risk in the context of axSpA is scarce [19].

Based on these data, in the present study, we aimed to explore the implication of vaspin, at the genetic and serological level, in subclinical atherosclerosis and CV risk in a large cohort of axSpA patients.

## Methods

### Patients

All the experiments involving humans and human blood samples were carried out in accordance with the approved guidelines and regulations, according to the Declaration of Helsinki.

All the patients included in this study belong to the *AtheSpAin* cohort, a Spanish multicentre cohort to study atherosclerosis in axSpA. In this regard, 510 Spanish patients who fulfilled the Assessment of SpondyloArthritis international Society classification criteria for axSpA [20] were recruited for this study at Hospital Universitario Marqués de Valdecilla (Santander), Hospital Comarcal de Laredo (Laredo), Hospital Universitario de Canarias (Santa Cruz de Tenerife), Hospital Universitario de Gran Canaria Dr. Negrín (Las Palmas de Gran Canaria), Hospital Universitario Reina Sofía (Córdoba), Hospital Universitario de La Princesa (Madrid), Hospital General Universitario de Elda (Alicante), Hospital General Universitario de Ciudad Real (Ciudad Real), Hospital Universitario La Paz (Madrid), and Hospital Universitario Basurto (Bilbao). None of them had diabetes mellitus or chronic kidney disease.

Data on sex, age, body mass index, blood pressure, total cholesterol, high-density lipoprotein (HDL)-cholesterol, low-density lipoprotein (LDL)-cholesterol and triglycerides at the time of study, as well as history of traditional CV risk factors (smoking, obesity, dyslipidemia, and hypertension) were collected. Obesity, dyslipidemia, and hypertension were defined as previously described [4]. In particular, obesity was defined if body mass index (calculated as weight in kilograms divided by height in squared meters) was ≥30. Routine laboratory parameters such as C-reactive protein (CRP) and erythrocyte sedimentation rate (ESR) were assessed at the time of the study. The main demographic, clinical, laboratory, and CV disease-related characteristics of patients are displayed in Table 1.

**Table 1:** Demographic, clinical, laboratory, and cardiovascular disease-related characteristics in patients with axial spondyloarthritis

Variable	axSpA (*n* = 510)
Men/women, *n*	360/150
Age (years), mean ± SD	48.8 ± 12.4
Age at axSpA diagnosis (years), mean ± SD	35.8 ± 11.4
C-reactive protein (mg/L), mean ± SD	5.7 ± 9.8
Erythrocyte sedimentation rate (mm/1st hour), mean ± SD	10.9 ± 16.2
Bath Ankylosing Spondylitis Disease Activity Index, mean ± SD	3.7 ± 2.3
Ankylosing Spondylitis Disease Activity Score, mean ± SD	2.2 ± 1.0
Bath Ankylosing Spondylitis Functional Index, mean ± SD	3.4 ± 2.6
HLA-B27 status, %	78.0
Peripheral synovitis, %	37.7
Hip involvement, %	20.4
Enthesitis, %	29.2
Extra-articular manifestations*, %	36.5
Syndesmophytes, %	43.5
History of classic cardiovascular risk factors, %	
Smoking	52.0
Obesity	22.4
Dyslipidemia	32.0
Hypertension	26.6
Body mass index (kg/m^2^), mean ± SD	27.2 ± 5.0
Systolic blood pressure (mm Hg), mean ± SD	129.1 ± 17.6
Diastolic blood pressure (mm Hg), mean ± SD	80.3 ± 11.2
Total cholesterol (mg/dL), mean ± SD	191.2 ± 39.4
HDL-cholesterol (mg/dL), mean ± SD	54.2 ± 16.5
LDL-cholesterol (mg/dL), mean ± SD	115.4 ± 32.6
Triglycerides (mg/dL), mean ± SD	122.7 ± 79.2
Atherogenic index (total cholesterol/HDL), mean ± SD	3.8 ± 1.2
Atherogenic index ≥4, %	36.5
Carotid IMT (mm), mean ± SD	0.646 ± 0.144
Carotid plaques, %	28.8

*axSpA* axial spondyloarthritis, *HDL* high-density lipoprotein, *IMT* intima-media thickness, *LDL* low-density lipoprotein, *SD* standard deviation. *Including anterior uveitis, psoriasis, and/or inflammatory bowel disease

Data shown in this table refer to values at the time of the study

Peripheral blood samples were collected in the fasting state from all the patients at the time of recruitment.

### Carotid US study

A carotid US study was performed in all the patients to assess the presence of abnormal carotid intima-media thickness (cIMT) values in the common carotid artery as well as the presence of focal plaques in the extracranial carotid tree (as surrogate markers of subclinical atherosclerosis), as previously reported [8].

### Serum vaspin assay

A commercial enzyme-linked immunosorbent assay kit was used to measure serum vaspin levels in axSpA patients (DY4410, DuoSet® ELISA, R&D Systems, Minneapolis, MN, USA) according to the manufacturer’s instructions. All samples were analyzed in duplicate and quantified relative to a standard curve, using 4-parameter logistic regression.

### Vaspin genotyping

Deoxyribonucleic acid of patients was obtained from peripheral blood using standard procedures. All of them were genotyped for *vaspin* rs2236242 (T/A) and rs35262691 (T/C) polymorphisms, previously associated with CV risk factors and/or reported as functional gene variants [21, 22]), using pre-designed TaqMan probes (C___2786211_1_ and C___7854490_10, respectively). Additionally, the rs7159023 (G/A) polymorphism was assessed with a pre-designed TaqMan probe (C__29386750_10). This polymorphism is in complete linkage disequilibrium (*r*^2^ = 1) with rs77060950 (G/T), also previously associated with serum vaspin levels [23]. Genotyping was performed in a QuantStudio™ 7 Flex Real-Time polymerase chain reaction system, according to the conditions recommended by the manufacturer (Applied Biosystems, Foster City, CA, USA). Negative controls and duplicate samples were included to check the accuracy of the genotyping.

### Statistical analysis

The Shapiro-Wilk normality test was performed and showed that serum levels of vaspin were not normally distributed in our cohort. Accordingly, these data were log transformed for the statistical analysis. The association of serum levels of vaspin with categorical and continuous variables was assessed by linear regression and Pearson’s partial correlation coefficient (*r*), respectively. The association of serum levels of vaspin with carotid plaques was tested by logistic regression, while the correlation between serum levels of vaspin and cIMT values was performed via estimation of the Pearson partial correlation coefficient (*r*). In all the cases, adjustment was performed for potential confounding factors: sex, age at the time of the study, and classic CV risk factors (smoking, obesity, dyslipidemia, and hypertension).

The *vaspin* rs2236242, rs7159023, and rs35262691 genotype data were checked for deviation from Hardy-Weinberg equilibrium (HWE). The link between genotypes, alleles, or haplotypes with serum levels of vaspin was tested by linear regression, adjusting for the potential confounding factors abovementioned. The relationship between genotypes, alleles, or haplotypes and carotid plaques was tested using logistic regression, while the association with cIMT values was evaluated by ANOVA, in both cases adjusting for potential confounding factors. In all the genetic analyses, the most frequent genotype, allele, and haplotype of *vaspin* rs2236242, rs7159023, and rs35262691 were used as reference.

Data were expressed as mean ± standard deviation (SD) for continuous variables, and number of individuals (*n*) or percentage (%) for categorical variables. Statistical significance was defined as *p* values ≤0.05, and all analyses were performed using STATA® v. 11.1 statistical software (Stata Corp, College Station, TX, USA).

## Results

### Relationship of serum vaspin with demographic features, CV risk factors, markers of inflammation and disease activity, and other axSpA features

**Fig. 1:**
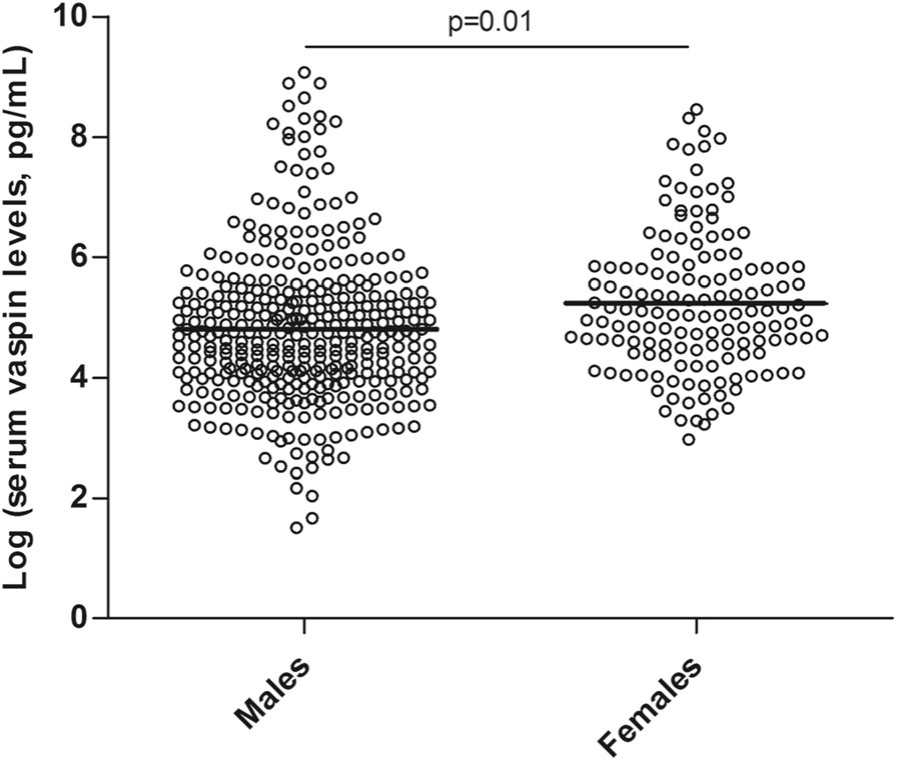
Scatter plot of log transformed serum vaspin levels in male and female axSpA patients. Vaspin levels were log transformed for statistical analysis, given that they were not normally distributed. Individual data points are shown, with means of each group indicated by horizontal lines

Serum vaspin levels were higher in female patients when compared to male patients (*p* = 0.01, Fig. 1).

**Fig. 2:**
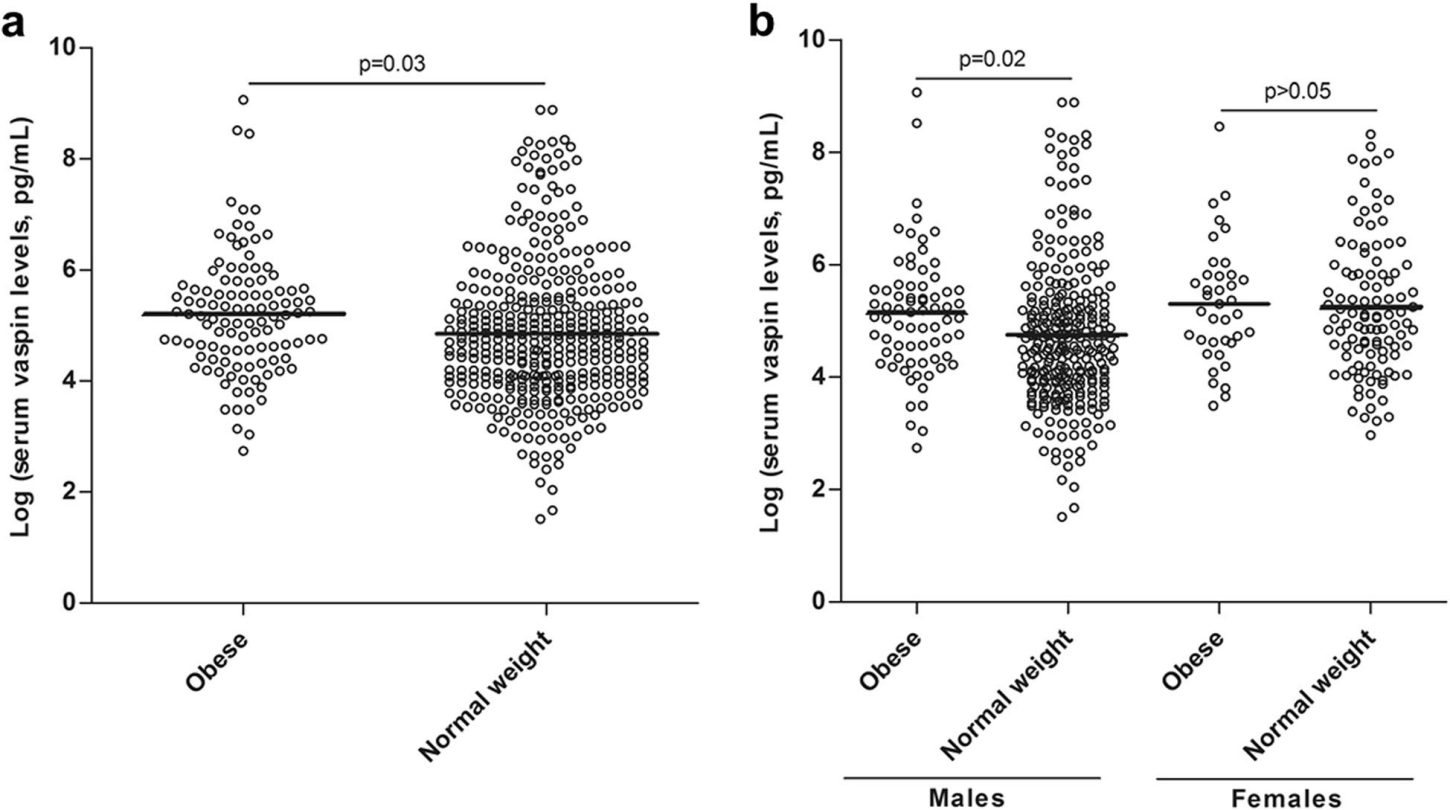
Scatter plot of log transformed serum vaspin levels in axSpA patients according to their obesity status: **a** in the whole cohort and **b** stratified by sex. Vaspin levels were log transformed for statistical analysis, given that they were not normally distributed. Individual data points are shown, with means of each group indicated by horizontal lines

When axSpA patients were stratified according to obesity, we disclosed that serum vaspin levels were higher in obese individuals when compared to those with normal weight (*p* = 0.03, Fig. 2a). Interestingly, when the association between serum vaspin levels and obesity was assessed stratifying by sex, we observed that this result only remained significant in male patients (*p* = 0.02), while it was not statistically significant in female patients (*p* > 0.05) (Fig. 2b).

No statistically significant results were obtained regarding the potential association of serum levels of vaspin with other CV risk factors including smoking status, dyslipidemia, hypertension, systolic and diastolic blood pressure, total cholesterol, HDL-c and LDL-c, triglycerides, and atherogenic index (*p* > 0.05). Similar results were obtained regarding markers of inflammation (CRP and ESR) or disease activity (Bath Ankylosing Spondylitis Disease Activity Score, Ankylosing Spondylitis Disease Activity Score and Bath Ankylosing Spondylitis Functional Index). Likewise, no association was observed between serum vaspin levels and other clinical features of axSpA: HLA-B27 status, peripheral synovitis, hip involvement, enthesitis, extra-articular manifestations, and syndesmophytes (*p* > 0.05).

### Genotype, allele, and haplotype distribution of rs2236242, rs7159023, and rs35262691

Genotyping success was greater than 97%. *Vaspin* rs2236242, rs7159023, and rs35262691 genotype distribution were in HWE (*p* > 0.05). Genotype and allele frequencies of rs2236242, rs7159023, and rs35262691 were in agreement with the data of the 1000 Genomes Project for Europeans. The distribution of the genotypes, alleles, and haplotypes of the three *vaspin* polymorphisms assessed in axSpA patients is shown in Tables 2 and 3.

**Table 2:** *Vaspin* rs2236242, rs7159023, and rs35262691 genotype and allele distribution in axial spondyloarthritis patients, and its influence on serum vaspin levels

Polymorphism	Genotype/allele	% (*n*)	Serum vaspin (pg/mL) (mean ± SD)	*p* value
rs2236242	TT	40.2 (202)	463.78 ± 1105.27	–
	TA	45.5 (229)	366.94 ± 858.96	0.09
	AA	14.3 (72)	236.04 ± 459.16	**0.03**
	T	62.9 (633)	428.64 ± 1022.11	–
	A	37.1 (373)	315.73 ± 730.77	**0.02**
rs7159023	GG	98.0 (490)	333.75 ± 781.27	**–**
	GA	2.0 (10)	3275.77 ± 2504.91	**< 0.001**
	AA	0.0 (0)	–	**–**
	G	99.0 (990)	361.42 ± 858.62	**–**
	A	1.0 (10)	3275.77 ± 2504.91	**< 0.001**
rs35262691	TT	35.2 (174)	332.45 ± 674.99	**–**
	TC	47.6 (235)	370.47 ± 961.18	0.92
	CC	17.2 (85)	539.71 ± 1242.04	0.93
	T	59.0 (583)	347.89 ± 802.48	–
	C	41.0 (405)	442.45 ± 1089.45	0.97

*SD* standard deviation

Statistically significant results are highlighted in bold

**Table 3:** *Vaspin* rs2236242, rs7159023, and rs35262691 haplotype distribution in axial spondyloarthritis patients and its influence on serum vaspin levels

Haplotype* (rs2236242, rs7159023, rs35262691)	% (*n*)	Serum vaspin (pg/mL) (mean ± SD)	*p* value
TGT	52.2 (513)	349.90 ± 810.90	–
AGC	30.0 (295)	303.13 ± 707.02	0.08
TGC	10.0 (98)	654.86 ± 1495.25	**0.01**
AGT	6.7 (66)	273.39 ± 481.02	0.82

*SD* standard deviation

*Only the results obtained from the most representative haplotypes (frequency higher than 5%) are shown in this table

Statistically significant results are highlighted in bold

### Influence of rs2236242, rs7159023, and rs35262691 on serum levels of vaspin

We found that patients homozygous for the minor allele of rs2236242 (AA genotype) showed the lowest serum levels of vaspin when compared to those carrying the reference TT genotype (*p* = 0.03, Table 2). Patients bearing the TA genotype showed intermediate levels of vaspin, but such differences were not statistically significant (*p* > 0.05, Table 2). Consequently, the minor allele of rs2236242 (A) was linked to lower serum vaspin levels when compared to the T allele (*p* = 0.02, Table 2).

Regarding rs7159023, the minor allele of this polymorphism (A) was associated with drastically higher serum levels of vaspin than the most frequent allele (G), both at the genotypic and allelic level (*p* < 0.001, Table 2). In this regard, although the group of patients carrying the A allele was small, it was interesting to notice that this subgroup of patients presented features associated with a more severe disease, such as hip involvement and high functional limitation, as well as a high prevalence of traditional CV risk factors, including obesity, dyslipidemia, and hypertension (Supplementary Table 1).

As for rs35262691, no statistically significant association was observed between this polymorphism and serum vaspin levels (*p* > 0.05, Table 2).

When the three *vaspin* polymorphisms were combined conforming haplotypes, the TGC haplotype associated with higher serum levels of vaspin when compared to the reference haplotype, TGT (*p* = 0.01, Table 3). Regarding the other *vaspin* haplotypes with frequencies higher than 5%, no statistically significant results were obtained (*p* > 0.05, Table 3).

### Association of vaspin and surrogate markers of subclinical atherosclerosis

No statistically significant association was observed between vaspin and markers of subclinical atherosclerosis (presence of carotid plaques and abnormal cIMT values) at the genetic or serological level (*p* > 0.05).

## Discussion

CV disease is the main leading cause of death in axSpA. Thereby, in the present study, we aimed to evaluate the potential implication of vaspin in CV risk and atherosclerotic disease in axSpA, in the search of new non-invasive biomarkers of CV risk that may help to attain an accurate and early diagnosis of this comorbidity in axSpA.

Mounting evidence indicates that vaspin plays a relevant role in atherosclerosis, protecting against the progression of this process through reducing vascular inflammation and oxidative stress, among other functions [12]. In fact, recent findings by Sato et al. revealed that vaspin is highly expressed in human coronary atheromatous plaques, particularly in macrophage foam cells and vascular smooth muscle cells [24]. However, evidence linking vaspin with atherosclerotic disease in the general population and in different pathologies has been clouded with contrasting results [15, 19, 25–28]. In concordance with the results reported by Aust et al. and Ozgen et al. [27, 28], in our study, we did not find any statistically significant association between vaspin and carotid plaques or abnormal cIMT values as surrogate markers of subclinical atherosclerosis. The only previous report in axSpA was performed in 120 Chinese patients with ankylosing spondylitis and reported an association between flow-mediated dilation and serum vaspin levels. Nonetheless, they did not evaluate the presence of plaques or cIMT [19].

Noteworthy, in our study, we observed that serum levels of vaspin were increased in female patients when compared to male patients. Similar results were previously reported by other groups in diverse contexts [16, 29]. Accordingly, and in harmony with previous data, sex hormones and fat distribution seem to exert an effect on serum vaspin levels [29, 30]. Furthermore, in our study, we found a positive association between serum levels of vaspin and obesity. This is in accordance with previous studies in other diseases and in the general population [30–35]. These results support the compensatory role proposed for vaspin in metabolic disturbances, aimed to improve glucose metabolism and to reduce the inflammatory process associated with obesity and related disorders [12]. Moreover, when patients were stratified according to sex, the association between serum levels of vaspin and obesity only remained significant in male patients. This interesting result was also described by Choi et al. in individuals with and without metabolic syndrome [16]. Nevertheless, further studies should be done to shed light on this sex-specific association of obesity and vaspin levels.

Finally, the results obtained in our study show, for the first time, that serum vaspin levels in axSpA are modulated by different polymorphisms in its coding gene. In particular, we found that the minor allele of rs2236242 (A) was associated with lower serum levels of vaspin, which is in accordance with the results obtained by other groups in patients with type 2 diabetes mellitus and in the general population [21, 36]. Similarly, we also disclosed that patients bearing the minor allele of rs7159023 (A) showed drastically higher serum levels of vaspin, in line with the results obtained in a previous study performed by Teshigawara et al. in which rs77060950 (in complete linkage disequilibrium with rs7159023) was assessed in the general population [23]. Of note, our data suggest that the presence of the minor allele of rs7159023 is linked to more severe disease and high CV risk. However, given the low frequency of this allele, further studies are needed to replicate our findings. Interestingly, when the three polymorphisms assessed were combined conforming haplotypes, we disclosed that the patients with the TGC haplotype exhibited higher serum levels of vaspin than those bearing the reference haplotype (TGT). These findings may have important implications since growing evidence suggests that haplotypes can provide a more comprehensive picture of the implication of a certain gene in different diseases, making evident effects of polymorphisms that cannot be detected when tested individually [37].

## Conclusions

Our results revealed that vaspin is linked to CV risk factors that may influence on the atherosclerotic process in axSpA. Additionally, we disclosed that serum vaspin concentration is genetically modulated in a large cohort of patients with axSpA.

## Supplementary Information

**Additional file 1: Supplementary Table 1.** Demographic, clinical, laboratory, and cardiovascular disease-related characteristics in patients with axial spondyloarthritis carrying or not the A allele of *vaspin* rs7159023.

## Data Availability

All data generated or analyzed during this study are included in this published article.

## Abbreviations

axSpA: Axial spondyloarthritis
cIMT: Carotid intima-media thickness
CRP: C-reactive protein
CV: Cardiovascular
ESR: Erythrocyte sedimentation rate
HDL: High-density lipoprotein
HWE: Hardy-Weinberg equilibrium
LDL: Low-density lipoprotein
SD: Standard deviation
US: Ultrasound
