# Supplementary material for: Vaspin in atherosclerotic disease and cardiovascular risk in axial spondyloarthritis: a genetic and serological study
Source: Arthritis Res Ther. 2021 Apr 13;23:111. doi: 10.1186/s13075-021-02499-7 (PMC8042971; doi:10.1186/s13075-021-02499-7)
Supplement: Supplementary file 1 — Additional file 1: Supplementary Table 1. Demographic, clinical, laboratory, and cardiovascular disease-related characteristics in patients with axial spondyloarthritis carrying or not the A allele of vaspin rs7159023. [file 13075_2021_2499_MOESM1_ESM.docx]

Additional file 1: **Supplementary Table 1.** Demographic, clinical, laboratory, and cardiovascular disease-related characteristics in patients with axial spondyloarthritis carrying or not the A allele of *vaspin* rs7159023.

|  | | *vaspin* rs7159023 | |
| --- | --- | --- | --- |
| Variable | | Non-carriers of A allele (n=490) | Carriers of A allele (n=10) |
| Men/Women, n | | 343/147 | 7/3 |
| Age (years), mean ± SD | | 49.0 ± 12.4 | 44.3 ± 12.0 |
| Age at axSpA diagnosis (years), mean ± SD | | 35.9 ± 11.4 | 34.8 ± 11.0 |
| C-reactive protein (mg/L), mean ± SD | | 5.8 ± 9.9 | 6.1 ± 5.4 |
| Erythrocyte Sedimentation Rate (mm/1st hour), mean ± SD | | 11.0 ± 16.5 | 7.1 ± 7.7 |
| Bath Ankylosing Spondylitis Disease Activity Index, mean± SD | | 3.7 ± 2.3 | 4.0 ± 2.9 |
| Ankylosing Spondylitis Disease Activity Score, mean± SD | | 2.2 ± 1.0 | 2.8 ± 0.9 |
| Bath Ankylosing Spondylitis Functional Index, mean ± SD | | 3.4 ± 2.6 | 5.3 ± 2.8 |
| HLA-B27 status, % | | 77.8 | 77.8 |
| Peripheral synovitis, % | | 37.8 | 50.0 |
| Hip involvement, % | | 19.6 | 70.0 |
| Enthesitis, % | | 29.2 | 50.0 |
| Extra-articular manifestations*, % | | 37.0 | 20.0 |
| Syndesmophytes, % | | 43.9 | 30.0 |
| History of classic cardiovascular risk factors, % | |  |  |
|  | Smoking | 51.8 | 70.0 |
|  | Obesity | 22.3 | 50.0 |
|  | Dyslipidaemia | 31.6 | 50.0 |
|  | Hypertension | 26.9 | 40.0 |
| Body mass index (kg/m^2^), mean ± SD | | 27.2 ± 5.0 | 30.1 ± 5.4 |
| Systolic blood pressure (mm Hg), mean ± SD | | 129.1 ± 17.8 | 137.1 ± 13.3 |
| Diastolic blood pressure (mm Hg), mean ± SD | | 80.3 ± 11.2 | 84.8 ± 12.1 |
| Total cholesterol (mg/dL), mean ± SD | | 191.1 ± 39.6 | 182.4 ± 38.9 |
| HDL-cholesterol (mg/dL), mean ± SD | | 54.3 ± 16.7 | 46.4 ± 10.1 |
| LDL-cholesterol (mg/dL), mean ± SD | | 115.5 ± 32.5 | 98.0 ± 35.8 |
| Triglycerides (mg/dL), mean ± SD | | 123.0 ± 80.1 | 136.6 ± 65.9 |
| Atherogenic index (total cholesterol/HDL), mean ± SD | | 3.8 ± 1.2 | 4.0 ± 0.9 |
| Atherogenic index ≥4, % | | 36.4 | 37.5 |
| Carotid IMT (mm), mean ± SD | | 0.646 ± 0.139 | 0.662 ± 0.355 |
| Carotid plaques, % | | 29.3 | 22.2 |
| Serum vaspin levels (pg/mL) | | 333.8 ± 781.3 | 3275.8 ± 2504.9 |

axSpA: Axial spondyloarthritis; HDL: High-Density Lipoprotein; IMT: Intima-Media Thickness; LDL: Low-Density Lipoprotein; SD: Standard Deviation. *Including anterior uveitis, psoriasis and/or inflammatory bowel disease.

Data shown in this table refer to values at the time of the study.
